# Editorial: New technologies for mechanical heart failure devices on the horizon: a non-distant future

**DOI:** 10.3389/fcvm.2024.1442164

**Published:** 2024-07-15

**Authors:** Massimo Bonacchi, Beatrice Bacchi, Francesco Cabrucci, Aleksander Dokollari, Rakesh Arora, Fabio Miraldi

**Affiliations:** ^1^ Cardiac Surgery F.U., Experimental and Clinical Medicine Department, University of Florence, Firenze, Italy; ^2^ Department of Cardiac Surgery, Lankenau Institute for Medical Research, Main Line Health, Wynnewood, PA, United States; ^3^ Department of Cardiac Surgery, University of Manitoba, Winnipeg, MB, Canada; ^4^ Department of Cardiac Surgery, Sapienza University of Rome, Rome, Italy

**Keywords:** mechanical circulatory support, mechanical circulatory devices, heart failure, LVAD (left ventricular assist device), impella

**Editorial on the Research Topic** New technologies for mechanical heart failure devices on the horizon: a non-distant future

The landscape of heart failure treatment is on the brink of a revolutionary transformation, thanks to cutting-edge advancements in mechanical heart failure devices. As the global burden of cardiovascular diseases continues to rise, with heart failure affecting over 26 million people worldwide, the need for innovative and effective solutions has never been more critical. Emerging technologies promise to enhance the quality of life for patients and reduce the mortality associated with this debilitating condition.

In this Research Collection, many aspects of recent advances in Heart Failure Treatment and mechanical circulatory support (MCS) have been collected.

Guidelines-directed medical therapy (GDMT), alone, in this setting of patients, is usually ineffective due to patients' compliance, gaps in implementation, inadequate follow-up or monitoring therapy, and lack of awareness or knowledge of the latest guidelines. The first strategy to improve this setting is undoubtedly the encouragement of quality-improvement strategies. Romero et al. suggested a titration clinic assisted with remote monitoring assessing the impact of the implementation of GDMT and achieving a relatively high patient compliance (85%) (Romero et al.).

Despite this and other improvements in pharmacology therapy and quality, many patients experience progressive and inevitable deterioration of their HF status due to a lack of adequate patient selection towards an upgraded therapy and critical timing.

MCS devices have been at the forefront of supporting daily life and cardiac surgery interventions of patients with advanced heart failure shaping (1), shaping the new concept of “protected heart surgery”. MCS, and in particular the Impella pump, in fact, offers an alternative and comprehensive treatment option for patients with severe heart failure and mitral valve disease who are not eligible for a transcatheter approach but in need of concomitant surgery (Osswald et al.). As a matter of fact, the Impella pump could also support patients with HFrEF undergoing life-saving surgical procedures, such as in cancer treatment, vascular surgery, or as a bridging device until the implantation of a left ventricular assist device (Guarracini et al.). The Impella pump, apart from its hemodynamic properties, provides an excellent bridge-to-recovery device without the disadvantages of postoperative patient immobilization or the need for extracorporeal circulation. Among the various elements of ERAS, in fact, together with the hemodynamic optimization, a faster patient recovery in the immediate postoperative period represents a cornerstone.

Despite their life-saving capabilities, current MCS devices are not without limitations, including complications like infection, thrombosis, and the need for regular maintenance. Therefore, the monitoring and management of adverse events associated with MCS are primary concerns for cardiologists and cardiothoracic surgeons (2). Over time, both clinical and benchtop approaches, together with new technologies as well as increased sophistication of standard technologies evolved to assess MCS. Wilson et al. reviewed several novel physical or digital technologies to evaluate the performance of LVAD (Wilson et al.). Despite these new techniques having been tremendously developed in recent years, the translation to the clinical scenario and the validation of their accuracy and utility in LVAD patients has still to be validated. In this setting a promising new echocardiographic tool with digital particle image velocimetry analysis creating 2D velocity field measurements of cardiac is being developed (Wilson et al.).

Another important aspect of MCS is the expense associated with the whole management of these devices. The cost considerations for MCS are, in fact, multifaceted and substantial, influencing both healthcare providers and patients. Despite the high costs, MCS devices can offer considerable benefits in terms of prolonged survival and improved quality of life for patients with advanced heart failure. However, cost-benefit analyses, incorporating both direct and indirect costs, are crucial to evaluate the overall value and justification of these devices in clinical practice (Fortunato et al.).

Therefore, while the future of mechanical heart failure devices looks promising, several clinical and practical challenges might still need to be addressed. Collaboration between researchers, medical professionals, regulatory bodies, and industry stakeholders will be essential to overcome everyday clinical practice obstacles.

The horizon of mechanical heart failure devices is bright, with innovations poised to transform the treatment landscape. Novel technologies are converging to create more effective, safer, and patient-friendly solutions. As we move forward, a concerted translational collaboration will be crucial to bring these technologies to those who need them most, heralding a new era of hope and improved outcomes for heart failure patients worldwide.
